# Histone Methyltransferases AcDot1 and AcRmtA Are Involved in Growth Regulation, Secondary Metabolism, and Stress Response in *Aspergillus carbonarius*

**DOI:** 10.3390/toxins17040196

**Published:** 2025-04-12

**Authors:** Angelo Agnusdei, Adrián González-García, Donato Gerin, Stefania Pollastro, Francesco Faretra, Luis González-Candelas, Ana-Rosa Ballester

**Affiliations:** 1 Department of Soil, Plant and Food Sciences, University of Bari Aldo Moro, Via Giovanni Amendola, 165/A, 70126 Bari, Italy; angelo.agnusdei@uniba.it (A.A.); stefania.pollastro@uniba.it (S.P.); francesco.faretra@uniba.it (F.F.); 2 Institute of Agrochemistry and Food Technology, Spanish Council for Scientific Research (IATA-CSIC), Calle Catedrático Agustín Escardino 7, 46980 Paterna, Valencia, Spain; adgongar@iata.csic.es (A.G.-G.); luis.gonzalez@iata.csic.es (L.G.-C.)

**Keywords:** histone post-translational modifications (HPTMs), ochratoxin A (OTA), virulence, oxidative stress, osmotic stress

## Abstract

Histone post-translational modifications (HPTMs) can affect gene expression by rearranging chromatin structure. Between these, histone methylation is one of the most studied in filamentous fungi, and different conserved domains coding for methyltransferase were found in *Aspergillus* spp. genomes. In this work, the role of the histone methyltransferases AcDot1 and AcRmtA in the mycotoxigenic fungus *Aspergillus carbonarius* was investigated, obtaining knockout or overexpression mutants through *Agrobacterium tumefaciens*-mediated transformation (ATMT). *A. carbonarius* is responsible for grape-bunch rot, representing the major source of ochratoxin A (OTA) contamination on grapes. In vivo conditions, the deletion of *Acdot1* or *AcrmtA* resulted in upregulation of growth when the isolates were cultivated on a minimal medium. The influence of *Acdot1* on the OTA biosynthesis was differently affected by culture conditions. On rich media, an increase in OTA accumulation was observed, while on minimal medium, lower OTA concentrations were reported. The deletion of *AcrmtA* always resulted in lower OTA accumulation. However, the expression of OTA biosynthesis genes was regulated by both histone methyltransferases. Of the six analyzed OTA genes, three of them showed altered expression in the knockout mutants, and *otaB* and *otaR1* were common between both mutants. Furthermore, both AcDot1 and AcRmtA play a role in oxidative stress response, induced by 1 mM hydrogen peroxide, by modulating growth, conidiation and OTA biosynthesis. Neither the deletion nor the overexpression of the *Acdot1* or *AcrmtA* affected virulence, while both the sporulation and OTA production were negatively affected in vivo by the deletion of *AcrmtA.*

## 1. Introduction

*Aspergillus carbonarius* (section *Nigri*) is one of the most important producers of OTA [1], a mycotoxin common contaminant of a wide range of foods such as wine, in which Zimmerli and Dick [2] first reported OTA, and other products derived from grapes, such as vinegar [3] and raisins [4]. The consumption of wine represents a high-risk exposure for the population of the Mediterranean area, such as southern Italy [5] and Spain [6]. On grapes, *A. carbonarius* is the main source of OTA contamination, also due to the high percentage of its isolates able to produce the mycotoxin [7], and the contamination is more frequent on grapes from warmer climates [8] and on red wines [9]. The toxicological risk for humans [10] and the poor availability of detoxification systems without an impact on the organoleptic quality led to the need for a better comprehension of OTA biosynthesis regulation in *A. carbonarius*, also to develop new sustainable control strategies.

The OTA biosynthetic pathway has been thoroughly investigated in the *Aspergillus* genera, and a consensus OTA biosynthetic pathway was described by Wang et al. [11] in *A. ochraceus*. Similarly, the key genes *otaA*, *otaB*, *otaC*, *otaD*, *otaY*, and *otaR1* were characterized in *A. carbonarius* [12,13,14,15]. The availability of the *A. carbonarius* genome (https://mycocosm.jgi.doe.gov/Aspca4/Aspca4.home.html, accessed on 1 March 2023) also allowed the identification of clusters related to response to stress and environmental stimuli, such as pH, water activity, light wavelengths, and temperature [16,17,18,19]. However, the secondary metabolism in filamentous fungi is subjected to a complex multi-tier regulation that involves environmental stimuli, signal transduction pathways, and regulation at transcriptional, translational, and epigenetic level [20]; therefore, the comprehension of the relationships between all these factors still requires further effort. In the last decade, histone post-translational modifications (HPTMs) have been suggested as an important mechanism in transcriptional regulation, influencing the phenotype also in response to environmental factors [21,22,23]. In *Aspergillus* genera, several works demonstrated how changes in chromatin conformation can regulate not only primary metabolism, but also virulence and secondary metabolism [24,25,26,27,28,29]. Among the HPTMs, histone methylation is one of the most studied, and different conserved domains have been identified in *Aspergillus* spp. Genomes [30]. Between these, three protein arginine methyltransferases (PRMTs; RmtA, RmtB, and RmtC) have been identified in *A. nidulans*, in which the RmtA domain is responsible of the methylation of histone H4 arginine 3 and other non-histone proteins, modulating the oxidative stress response [31]. Its homologous in *A. flavus* acts as a repressor of asexual development, under-regulating the genes *brlA*, *abaA*, and *wetA*, it upregulates the biosynthesis of aflatoxin B1 and it is involved in stress response [32,33]. The conserved domain Dot1 is reported to catalyze the mono-, di-, and trimethylation of the nucleosome core on lysine 79 of histone H3 (H3K79), acting on cell cycle regulation, and in DNA damage response [34]. In *Penicillium oxalicum*, PoDot1 affects conidiation by regulating the transcription of key regulators (BrlA, FlbC, and StuA) and it is required in normal hyphae septum and branch, while in *A. flavus* Dot1 regulates conidiation, fungal growth, sclerotia, and aflatoxin B1 production and affects the response to multiple stresses [35]. In this work, the role of two histone methyltransferases (AcDot1 and AcRmtA), selected for their genetic proximity to the already characterized *A. flavus*, was investigated in *A. carbonarius*. Through *Agrobacterium tumefaciens*-mediated transformation (ATMT), three deletion mutants (AcΔ*dot1* and AcΔ*rmtA*) and three overexpression mutants (AcOE*dot1* and AcOE*rmtA*) per both genes were obtained and screened in vitro for vegetative growth, conidiation, OTA production, and response to osmotic and oxidative stresses. Furthermore, their virulence was assessed in vivo on grape berries.

## 2. Results

### 2.1. Phylogenetic Analysis and Generation of A. carbonarius Mutants

To characterize the homolog of the *A. flavus* histone methyltransferase Dot1 [35] in *A. carbonarius*, BlastP analyses were performed using the MycoCosm database [36] with the amino acid sequence of *A. flavus* Dot1 (Protein ID: EED49233, Gene ID: AFLA_093140) as a query. Aspca4|681903, encoding a 501 aa protein, was identified in *A. carbonarius*, sharing a 77.7% overall identity with *A. flavus* Dot1. *Acdot1* consists of two exons and functional analysis performed using InterPro resources showed that the protein possesses the histone–lysine N-methyltransferase DOT1 domain (InterPro ID: IPR025789) responsible for the trimethylation of histone H3 to form H3K79me3.

**Figure 1:**
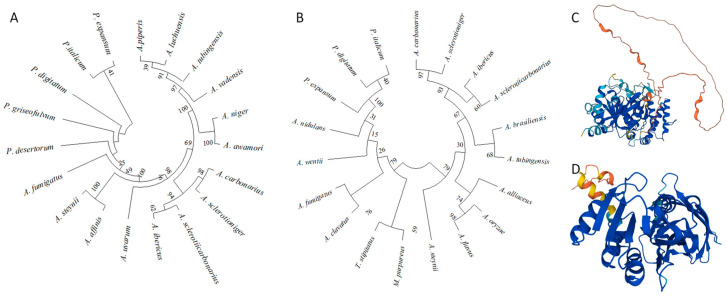
Analysis of the proteins of interest. Phylogenetic trees on: (**A**) Acdot1 and (**B**) AcrmtA, constructed by MEGA (version 11) on 19 protein sequences. Bootstraps values are calculated on 1000 trees. Three-dimensional structures of (**C**) Acdot1 and (**D**) AcrmtA, obtained from the Uniprot database (Acdot1 entry: A0A1R3RSP1; AcrmtA entry: A0A1R3RRD9). Distinct colors indicate different values of model confidence, expressed in per-residue confidence score (pLDDT): blue = very high (pLDDT > 90); light blue = confident (90 > pLDDT > 70); yellow = low (70 > pLDDT > 50); orange = very low (pLDDT < 50).

The Aspca4|1103151 is the orthologue of the AflRmtA (Protein ID: EED49233) [32] identified by BlastP on the *A. carbonarius* ITEM 5010_20210312_filtered model protein MycoCosm database with an overall identity of 95.6%. The corresponding *AcrmtA* gene consisted of seven exons and codes for an arginine N-methyltransferase protein (InterPro domain IPR025799), characterized by the presence of the methyltransferase domain 25 (InterPro domain IPR041698). The evolutionary relationship, analyzed by constructing phylogenetic trees from 19 protein sequences by MEGA 11, demonstrated that the two proteins are conserved within the genus *Aspergillus* (Figure 1A,B).

The USER-Friendly cloning strategy [37] allowed the obtainment of the gene replacement plasmids p507-H2-AcDot1 and p507-H2-AcRmtA (Figure S1A,B) and the plasmids p507-HE-AcDot1 and p507-HE-AcRmtA for random integration (Figure S1C,D). The knockout mutants Δ*dot1* and Δ*rmtA* were obtained by homologous recombination strategy (Figure S1E), while the overexpression mutants OE*dot1* and OE*rmtA* by random integration of the GOI preceded by the *gpdA* promoter (Figure S1F), as described by Frandsen et al. [37]. Three days after *A. tumefaciens* co-inoculation with *A. carbonarius* conidia, transformant colonies were screened on selective media, and hygromycin resistant monosporic cultures were obtained. PCR analysis confirmed the correct insertion of the T-DNA in the genome of the mutants with the replacement of the gene of interest with the hygromycin-resistance cassette in the knockout mutants and the ectopic integration in the overexpression ones. Finally, to select knockout or overexpression mutants, the number of T-DNA was determined by quantitative real-time PCR (qPCR), and three mutants for each transformation were selected for further analysis (Table 1).

### 2.2. Characterization of Mutants

**Figure 2:**
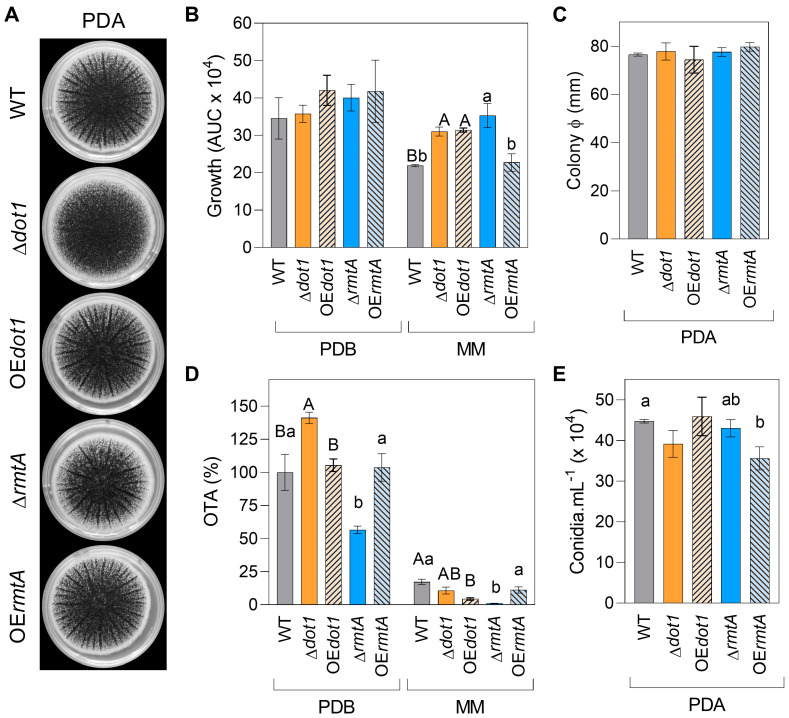
Phenotypic characterization of wild-type (WT, black bars), knockout mutants (Δ*dot1*, orange, and Δ*rmtA*, blue) and overexpression mutants (OE*dot1*, orange with black lines bars, and OE*rmtA*, blue with black lines bars). (**A**) Morphology of 7-day-old PDA cultures. (**B**) Growth area on PDB and MM was calculated after 6 days of growth at 24 °C. (**C**) Orthogonal colony diameter of 7-day-old PDA cultures. (**D**) OTA production determined by HPLC analysis after 6 days of growth on PDB and MM. (**E**) Conidiation was determined on three PDA plugs, from the middle of 7-day-old colonies. The values are the average of three different biological replicates, with their standard error. Different uppercase letters indicate statistically significant differences between WT, Δ*dot1* and OE*dot1* mutant, while different lowercase letters indicate differences between WT, Δ*rmtA* and OE*rmtA* mutants, determined by one-way ANOVA followed by Tukey’s HSD test with *p* ≤ 0.05.

Phenotypic characterization of the different strains was performed in PDB and MM broth, and on the solid medium PDA (Figure 2A). Only Δ*dot1* colonies showed macro-morphological differences on PDA at 7 DAI with a more diffuse and uniform colony and without pronounced radiating structures. The Δ*dot1*, OE*dot1*, and Δ*rmtA* mutants showed a significant increase in the AUC at 6 DAI when grown on MM (41%, 43% and 61%, respectively), while no statistically significant differences were observed between the vegetative growth of the mutants and the WT when the strains were grown on PDB and PDA (Figure 2B,C). The role of the two methyltransferases on OTA secretion was strongly affected by the growth media and the highest OTA productions was observed in PDB (Figure 2D). The deletion of *dot1* led to an increase in OTA production when the mutants were grown on PDB (41%), but not on MM, while the OE*dot1* mutants showed a reduction in OTA secretion on MM (−37%), but not in PDB. OTA production was strongly affected by the deletion of *rmtA* on both media (−43% on PDB and −93% on MM), while no statistically significant differences with respect to the WT were observed in OE*rmtA* mutants. Similar conidia production was observed for all the isolates on 7-day-old PDA cultures, except for OE*rmtA* mutants for which a slight reduction in conidiation (−17%) was reported (Figure 2E). Conidia from 4-day-old PDA cultures were stained with calcofluor white and observed under a microscope and no differences were observed between WT and the mutants on their morphology. The surface morphology of all the immature conidia was easily observed under epifluorescence (Figure S2). Moreover, no differences in conidia germinability (99–100%) and germ tube elongation of mutant isolates were observed after overnight incubation at 24 °C on AG plugs, confirming that neither Dot1 nor RmtA have an influence on the vitality of the conidia (Figure S3).

### 2.3. Gene Expression Analysis

**Figure 3:**
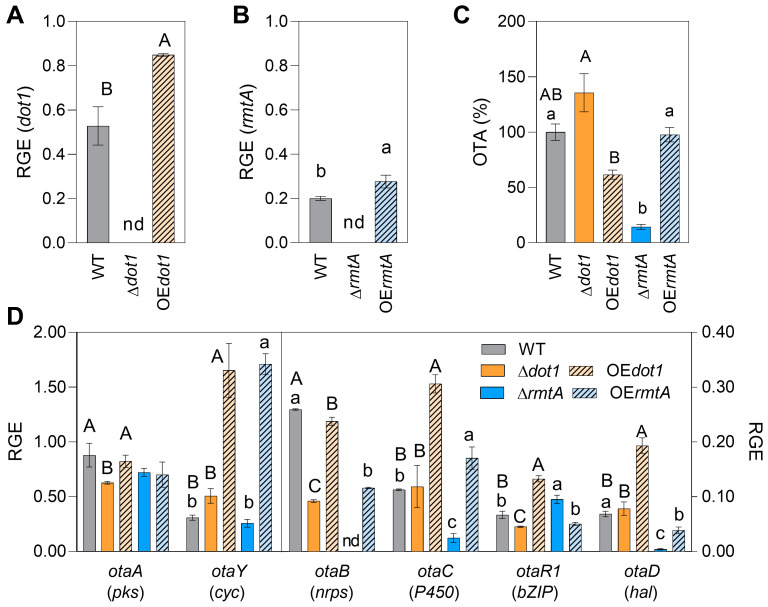
Relative gene expression values (RGE) of (**A**) *dot1*; (**B**) *rmtA*; (**D**) genes of OTA biosynthetic pathway in the wild-type (WT, black bars), knockout mutants (Δ*dot1*, orange, and Δ*rmtA*, blue) and overexpression mutants (OE*dot1*, orange with black lines bars, and OE*rmtA*, blue with black lines bars). cDNA was synthetized from 500 ng of total RNA obtained from 4-day-old cultures, grown in liquid MM in OTA-I conditions (OTA-I 25 °C in the dark, without shaking). (**C**) OTA production in liquid MM after 4 days of growth in OTA inducive conditions. The values are an average of three biological replicates with their standard error. Different uppercase letters indicate statistically significant differences between WT, Δ*dot1*, and OE*dot1* mutants, while different lowercase letters indicate differences between WT, Δ*rmtA*, and OE*rmtA* mutants, determined by one-way ANOVA, followed by Tukey’s HSD test with *p* ≤ 0.05. “nd” indicates non detected.

The gene expression study on *dot1* and *rmtA* confirmed the deletion and the overexpression of the GOI in the deletant and overexpressed mutants, respectively. After 4 days of incubation in OTA-I conditions, a complete downregulation of the target gene was observed in Δ*dot1* and Δ*rmtA* knockout mutants, while a significant overexpression of *dot1* (+61%) and *rmtA* (+38%) was reported for OE*dot1* and OE*rmtA* mutants, respectively (Figure 3A,B). To investigate the regulative role of the *dot1* and *rmtA* on OTA biosynthetic pathway, the expression level of the key genes of the OTA biosynthetic pathway was also investigated by RT-qPCR in the mutant isolates in comparation with the WT. A slight downregulation of *otaA*, *otaB* and *otaR1* genes was observed in Δ*dot1* mutants, while the *otaY*, *otaD* and *otaC* showed expression values like the WT. In Δ*rmtA*, although the expression of the *otaA* and *otaY* mutants were like WT, and the otaR1 resulted slightly up regulated, the expression of the *otaB*, *otaC*, *otaD* was strongly downregulated, explaining the reduction in OTA production observed in this mutant (Figure 3C). The overexpression of the *dot1* determined an upregulation of *otaY*, *otaC*, *otaR1* and *otaD* in the OE*dot1* mutants, while the expression of *otaA* was WT like and *otaB* showed a slight downregulation. In the OE*rmtA* mutants an increase in *otaY* and *otaC* expression was reported, while for the *otaA* and *otaR1* the expression values were WT like and for *otaB* and *otaD* were slightly downregulated (Figure 3D).

### 2.4. Response to Osmotic and Oxidtive Stresses

**Figure 4:**
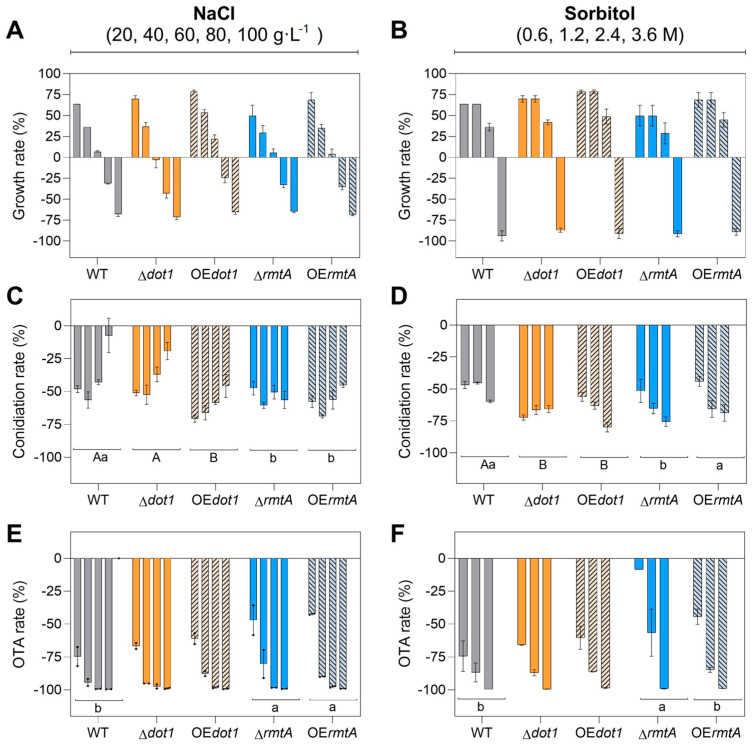
Response to osmotic stress of wild-type strain (WT, black bars), knockout mutants (Δ*dot1*, orange, and Δ*rmtA*, blue) and overexpression mutants (OE*dot1*, orange with black lines bars, and OE*rmtA*, blue with black lines bars). (**A**,**B**) The growth rate, (**C**,**D**) the conidiation rate and (**E**,**F**) the OTA production rate were evaluated growing the mutants and the WT on PDA supplemented with increasing concentrations of (**A**,**C**,**E**) NaCl (20, 40, 60, 80, and 100 g·L^−1^) or (**B**,**D**,**F**) D-sorbitol (0.6, 1.2, 2.4, and 3.6 M). The data shown are the mean of three biological replicates with their standard error. Different uppercase letters indicate statistically significant differences between WT, Δ*dot1* and OE*dot1* mutant, while different lowercase letters indicate differences between WT, Δ*rmtA* and OE*rmtA* mutants, determined by one-way ANOVA, followed by Tukey’s HSD test with *p* ≤ 0.05.

Similar growth trends were observed for all the strains when cultivated on PDA amended with increasing concentrations of NaCl or D-Sorbitol. The growth of all the strains was promoted by lower osmolyte concentrations and, an inhibition effect was observed starting from 80 g·L^−1^ of NaCl or 3.6 M D-sorbitol (Figure 4A). The osmotic stress negatively affected the conidiation in all the isolates. However, a reduction of 70% was observed in the conidiation rate of the OE*dot1* already in the presence of 20 g·L^−1^ of NaCl, while a reduction of 49% was observed for the WT. Increasing the concentration up to 80 g·L^−1^ of NaCl, a higher reduction in the conidiation rate, with respect to WT was observed also for Δ*rmtA*, OE*dot1* and OE*rmtA* mutants (−56%, −45%, −30%, respectively, versus −7% in the WT). Otherwise, 0.6 M of D-sorbitol determined a stronger inhibition of the conidiation rate for Δ*dot1* and OE*dot1* isolates (−75% and −56%, respectively) compared to the WT (−45%). Increasing the D-sorbitol concentrations, all the mutants showed higher susceptibility to the stress agent than the WT, and the inhibition values ranged from −57% (OE*rmtA*) to −67% (Δ*dot1*) at 1.2 M, and from −64% (OE*rmtA*) to −79% (OE*dot1*) at 2.4 M (Figure 4B). OTA secretion was negatively affected by osmotic stress in all the isolates; however, Δ*rmtA* and OE*rmtA* mutants showed a better tolerance to the lower doses of the osmolyte assessed. At 20 g·L^−1^ of NaCl, a reduction of 75% was observed in the WT, while 46% and 42% were the reductions observed for Δ*rmtA* and OE*rmtA* mutants, respectively. Similarly, in the presence of 0.6 M D-sorbitol, a reduction in OTA production by 14% was observed in the Δ*rmtA* mutants and 47% in the OE*rmtA*, compared to a 70% decrease observed in the WT. Increasing the osmolyte concentration, the OTA production was strongly affected in all strains, up to approximately 99% inhibition for the highest concentrations assessed (80 g·L^−1^ of NaCl or 3.6 M D-sorbitol) (Figure 4C).

**Figure 5:**
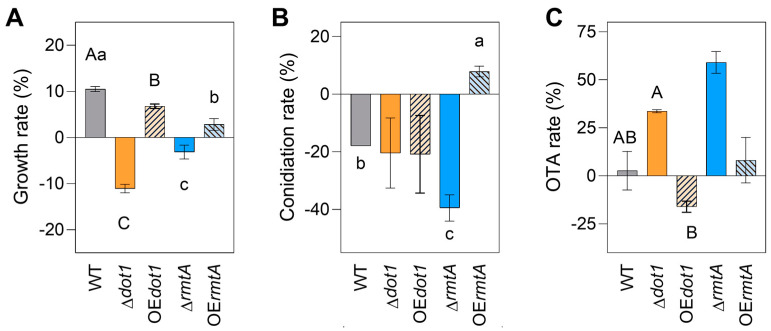
Response to oxidative stress of the wild-type (WT, black bars), knockout mutants (Δ*dot1*, orange, and Δ*AcrmtA*, blue) and overexpression mutants (OE*dot1*, orange with black lines bars, and OE*rmtA*, blue with black lines bars). The inhibition of (**A**) growth, (**B**) conidiation and (**C**) OTA production was evaluated growing the mutants and the WT on PDA supplied with 1 mM hydrogen peroxide (H_2_O_2_). Data shown are the mean of three biological replicates with their standard error. Different uppercase letters indicate statistically significant differences between WT, Δ*dot1* and OE*dot1* mutant, while different lowercase letters indicate differences between WT, Δ*rmtA* and OE*rmtA* mutants, determined by one-way ANOVA, followed by Tukey’s HSD test with *p* ≤ 0.05.

To evaluate the role of Dot1 and RmtA under oxidative stress response, the WT and the mutant isolates were grown on PDA supplemented with 1 mM hydrogen peroxide (H_2_O_2_). The oxidative stress inhibited the Δ*dot1* and Δ*rmtA* growth rates (−11% and −3.2%, respectively), while the growth of the WT and, to a lesser extent, the OE*dot1* and OE*rmtA* strains was slightly promoted by the presence of H_2_O_2_ (+10%, +7% and +2.8, respectively) (Figure 5A). In general, oxidative stress negatively affected the conidiation in all the mutants, except for the OE*rmtA* strains for which a slight increase in the conidiation rate was observed (+8%). However, in Δ*rmtA* isolates, the inhibitory effect of the oxidative agent on conidiation was significantly higher (−40%), reducing the number of conidia by 2-fold, compared to the WT (−17%) (Figure 5B). An increase in OTA production was observed in the presence of oxidative stress in the Δ*dot1* and Δ*rmtA* (+34 and +50%, respectively), while a slight reduction in OTA accumulation was observed (−16%) only in the OE*dot1* mutants (Figure 5C).

### 2.5. In Vivo Evaluations

**Figure 6:**
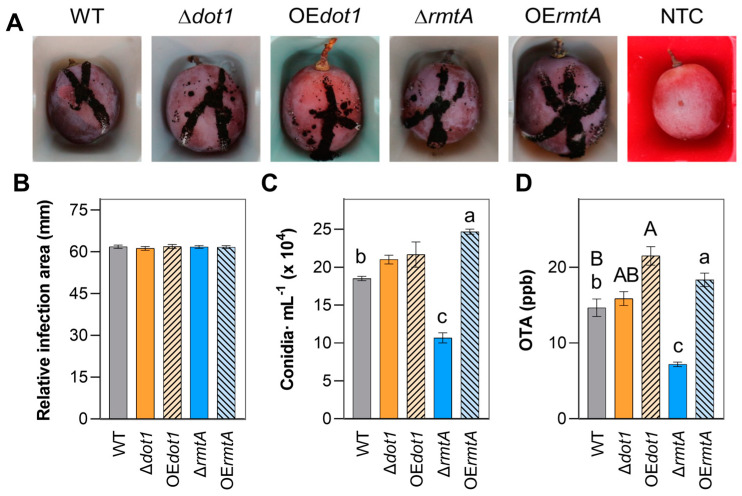
Virulence of the wild-type (WT, black bars), knockout mutants (Δ*dot1*, orange, and Δ*rmtA*, blue) and overexpression mutants (OE*dot1*, orange with black lines bars, and OE*rmtA*, blue with black lines bars) was evaluated in vivo on Sugra 53 berries inoculating three replicates of 4 berries with 5 µL of conidia suspension (1 × 10^4^ conidia∙mL^−1^) for each mutant. (**A**) Colonization of the berries 7 days after inoculation (DAI). (**B**) Relative area of developing lesions on berries was calculated normalizing the infection area to the area of the berries at 3 DAI. (**C**) Conidia production on berries at 7 DAI. (**D**) OTA contamination at 7 DAI. Data are the mean values of three replicates and their standard errors. Different uppercase letters indicate statistically significant differences between WT, Δ*dot1* and OE*dot1* mutants, while different lowercase letters indicate differences between WT, Δ*rmtA* and OE*rmtA* mutants, determined by one-way ANOVA analysis, followed by Tukey–Kramer or Tukey’s HSD test with *p* ≤ 0.05.

On ‘Sugra 53’, a similar colonization rate, expressed as relative infection area on the berries, was observed for all the isolates at 2, 3 and 4 DAI (Figure 6A,B). Δ*dot1* and OE*dot1* isolates were WT like in conidia production, while the deletion of *rmtA* inhibited the conidiation rate by 42% with respect to WT, and a slight increase was observed in the OE*rmtA* mutants (+32%) (Figure 6C). A statistically significant reduction in OTA accumulation (−50%) was observed in Δ*rmtA* isolate with respect to WT, while the overexpression of the *rmtA* resulted in OTA increasing (+25%). The deletion of *dot1* did not affect OTA production, while an increase in accumulation was observed in OE*dot1* isolates (46%).

## 3. Discussion

Histone post-translational modifications (HPTMs), such as acetylation, methylation, phosphorylation, ubiquitylation, or SUMOylation, can occur in the N-terminal tails or in the histone’s core [38]. Affecting the histone–DNA, histone–histone and histone–chaperone interactions [39]. These modifications can play an important role in gene expression by rearranging the chromatin structure [40], but also in DNA repair and damage response [41,42,43], in protein–protein interaction and protein functions [44,45]. HPTMs can directly regulate the chromatin structure, or act as binding sites for other non-histone proteins [46] with a chromodomain [47], frequently associated with metabolic pathways and strictly dependent on the availability of metabolites. In this way, histone modifications can be an important mechanism for nutrient sensing and environment adaption [48], representing a link between genotype and environment able to modify phenotypic expression [21,49,50,51]. The role of post-translational modifications has been extensively studied also in filamentous fungi [29,52,53,54], and several authors demonstrated a relationship between HPTMs and regulation of growth and pathogenesis [25,55,56], but also with secondary metabolism. In recent years, HPTMs have raised concerns for their regulatory activity of different cellular processes in *Aspergillus* [56,57], a genus including ubiquitous plant pathogens as well as several mycotoxins producers, and the effects of different histone’s alteration have been highlighted. In this work, the histone methyltransferase AcDot1, showing 77.7% identity with the Dot1 of *A. flavus*, and the arginine methyltransferase AcRmtA, showing 95.6%. identity with *AflRmtA* were identified in *A. carbonarius*, a filamentous fungus commonly associated with grapevine bunch rot, also reported as the main producer of OTA on grapes. ATMT knockout and overexpression mutants were obtained to study their role on primary and secondary metabolism and stress response. The in silico study showed that both Dot1 and RmtA are highly conserved within *Aspergillus* genera, always containing the histone–lysine N-methyltransferase DOT1 domain and the SAM binding domain, respectively, according to previous studies [33,58].

The DOT1 domain shows methyltransferase activity toward histone H3 Lys79 and has been widely studied on yeast, in which its role in nucleosome dynamics through histone exchange has been demonstrated. In vitro conditions, neither deletion nor overexpression of *Acdot1* affected the growth of the isolates on rich media, while on liquid minimal medium, both mutations positively influenced the growth (Figure 2), underlying the impact of the growth conditions on the role of AcDot1. In a previous work, Dot1 showed a positive effect on growth regulation in *A. flavus* when the fungus was grown on PDA [35], while in *Alternaria alternata*, *Aa*∆*dot* mutants and WT showed the same rate of vegetative growth, always on rich media [59]. The conidiation was not affected by either knockout or overexpression of *Acdot1*. In the model organism *A. nidulans* the histone methylation is involved in normal induction of secondary metabolism [56] as well as in *A. flavus*, where the production of aflatoxin B1 was severely impaired by *dot1* deletion [35]. Here, the deletion of *Acdot1* determined an upregulation of the OTA biosynthesis on liquid rich media, whereas the AcOE*dot1* isolates showed a WT-like phenotype. However, on minimal media the AcOE*dot1* mutants showed less amount of OTA, according to the slight under expression of the key gene *otaB* observed. The involvement of histone methylation and Dot1 in response to multiple stresses have been extensively studied in mammalian and yeast [34] and mutations affecting the Dot1 enzymatic activity can result into defects in response to multiple genotoxic stresses [60]. The vegetative growth of the Ac∆*dot1* isolates was found to be strongly compromised in the presence of oxidative stress induced by hydrogen peroxide (Figure 5), while in the presence of increasing osmotic stress the knockout mutants showed a growth rate like the WT (Figure 4). Conversely, the AcOE*dot1* isolates showed better tolerance to stresses, with growth rates like the WT. In terms of conidiation, the mutants showed the same production trend as the WT in the presence of oxidative stress, while the AcOE*dot1* isolates were more sensitive to both NaCl and D-sorbitol, showing a greater reduction in the number of conidia. OTA accumulation was found to be strongly influenced by oxidative stress. In the Ac∆*dot1* a significant increase in mycotoxin production was observed in the presence of hydrogen peroxide, while the overexpression of the gene determined a slight inhibition of OTA secretion. The role of Dot1 in modulating primary and secondary metabolism under stress conditions was previously reported for *A. flavus*, in which ∆*dot1* mutants showed less sensitivity to genotoxicity stress, cell damaging agents and oxidative stress than the WT [35]. Although the influence of Dot1 on the virulence of many plant pathogens has been reported [61,62], in vivo conditions, neither the deletion nor the overexpression of *Acdot1* resulted in changes in the virulence of *A. carbonarius*. The mutant isolates, in fact, showed a WT like berries’ colonization rates and conidiation trends. Only the overexpression of *Acdot1*, resulted in a slight increase in OTA accumulation at 4 DAI (Figure 6).

In *A. nidulans*, three conserved PRMTs, denominated RmtA, RmtB and RmtC were found to be involved in oxidation-reduction processes as well as transport and secondary metabolism [63]. RmtA is responsible for histone H4 arginine 3 methylation [57] and it has been related to stress response, virulence and secondary metabolism in different filamentous fungi [20,64,65]. Although several works on *Aspergillus* species did not report a role of RmtA on vegetative growth, the methyltransferase has been demonstrated as essential for the growth in *Neurospora crassa* [66]. In this work, in vitro conditions, the growth of AcΔ*rmtA* isolates was positively influenced only in liquid minimal medium, but not on rich media (Figure 2). The role of RmtA in conidiation was widely demonstrated in the literature. In *A. flavus*, the deletion of *rmtA* resulted in hyper conidiation, related to the overexpression of the regulatory genes *brlA* and *abaA*, while the overexpression of the gene resulted in a reduction in conidia production [32,33]. Similarly, in this work, the overexpression of the gene resulted in lower conidia production (Figure 2 and Figure 3), suggesting that AcRmtA negatively regulates the conidiation in *A. carbonarius*. Under all conditions assessed, the OTA production was strongly reduced in AcΔ*rmtA* isolates, similarly to what was reported for *A. flavus* [32,33]. The reduction in the mycotoxin secretion was correlated with a strong downregulation of the *otaB*, *otaC*, and *otaD* expression, although the regulator *otaR1* showed similar expression levels as the WT (Figure 3D). This would indicate a direct regulatory effect of AcRmtA on the genes of the biosynthetic cluster. Similarly, a role on the expression of key genes of aflatoxin biosynthesis (*aflR*, *aflC*, *aflK*) was observed in *A. flavus* [32]. The RmtA protein showed a crucial role in development and secondary metabolism in the presence of stress agents. In the presence of oxidative stress, the Δ*rmtA* isolates showed increased sensitivity to the stress agent with respect to WT, with reduced growth and conidiation, and higher production of OTA (Figure 5), according to what has been reported for *A. flavus*, as well as *Penicillium expansum* [33,64]. OTA production was found to be inhibited by osmotic stress, however in the presence of a moderate stress, dictated by the presence of 0.6 M sorbitol, a minor reduction in OTA accumulation was observed in the Δ*rmtA* isolates, compared to the WT, highlighting a lower sensitivity of the mutant to osmotic stress. Virulence towards berry colonization was not affected by deletion or overexpression of *rmtA*. The deletion of *rmtA* resulted in a reduction in the number of conidia on the berries (Figure 6), unlike what was previously reported for *A. flavus* on corn and peanuts [32], and in a reduction in OTA accumulation, confirming the in vitro observations and similarly to what was reported on maize for *A. flavus* [32], while the overexpression of the gene resulted in an increase in the production of the mycotoxin.

## 4. Conclusions

This work allowed us to investigate the role of the methyltransferases AcDot1 and AcRmtA in the regulation of primary metabolism, response to stress and OTA biosynthesis, representing a precursor in the study of epigenetic mechanisms in *A. carbonarius*. Although the protein sequences are highly conserved between different *Aspergillus* species, the regulative effects of the AcDot1 and AcRmtA have been shown to be strictly dependent on the species and the growth conditions, highlighting the role of methyltransferases in regulating gene expression in response to environmental stimuli. The results in vitro showed the involvement of AcDot1 and AcRmtA in vegetative growth, mycotoxin biosynthesis, and response to osmotic and oxidative stress in *A. carbonarius*. Furthermore, the in vivo assays confirmed the regulative role of the AcRmtA in conidiation and OTA biosynthesis, representing a new potential target for the control of *A. carbonarius* and OTA contamination risk.

## 5. Materials and Methods

### 5.1. Strains and Growing Conditions

*Aspergillus carbonarius* AC49 (CBS 144853), stored in the culture collection of the Department of Soil, Plant and Food Sciences of the University of Bari (Italy), and three selected mutant strains per each transformation, obtained in this study, were used. Fungal strains were stored as aqueous solution of 20% glycerol at −80 °C until use and routinely grown on Potato Dextrose Agar (PDA), Potato Dextrose Broth (PDB) (Difco-BD Diagnostics, Sparks, MD, USA), or Minimal Medium (MM) [13] in the dark at 28 ± 1 °C. Conidia suspensions were obtained by scrapping off the spores from the surface of PDA plates with a sterile spatula, resuspending in water, and titrated in a hemocytometer. *Escherichia coli* DH5α and *Agrobacterium tumefaciens* AGL-1 strains were stored in the collection of the Department of Food Biotechnology, Instituto de Agroquimica y Tecnología de Alimentos (IATA-CSIC, Valencia, Spain). *E. coli* DH5α transformed cells were grown on Luria–Bertani (LB, Bacto tryptone 10 g, yeast extract 5 g, NaCl 5 g, per liter) supplemented with 25 µg∙mL^−1^ of kanamycin, while *A. tumefaciens* AGL-1 was grown in LB supplemented with 20 µg∙mL^−1^ of rifampicin, 100 µg∙mL^−1^ of kanamycin, and 75 µg∙mL^−1^ of carbenicillin. The evaluations on conidia germination were conducted on agar glucose [AG; 20 g agar (Agar Granulated, Bacteriological Grade—Formedium, Ltd. Norfolk, UK), 20 g dextrose (D (+)-Glucose anhydrous, PanReac Química S.L.U., Barcelona, Spain), per liter].

### 5.2. Gene Analysis and Phylogenetic Studies

The *A. carbonarius* ITEM 5010 genome v.4.0 (https://mycocosm.jgi.doe.gov/Aspca4, accessed on 1 March 2023) was used as a reference for genetic analysis. The AcDot1 and AcRmtA proteins were identified by BlastP analysis by using as query the AfDot1 of *Aspergillus flavus* (Protein ID: EED49233; Gene ID:AFLA_093140), and the AflRmtA of *A. flavus* (Protein ID:EED48514; Gene ID:AFLA_127370) in the MycoCosm, The Fungal Genomics Resource, database [36]. The AcDot1 and AcRmtA protein sequences were then used as queries for BlastP analysis on the non-redundant protein sequences NCBI database (http://www.ncbi.nlm.nih.gov/BLAST/, accessed on 1 March 2023) to identify orthologue proteins in other fungi. Nineteen protein sequences belonging to *Aspergillus* spp., *Penicillium* spp., and other genera were aligned with the Muscle Algorithm, and phylogenetic trees were obtained with the maximum likelihood method through MEGA (version 11.0.13) [67].

### 5.3. Obtaining A. carbonarius Mutants

All the primers used in this work (Table 2) were designed using Primer3Plus (version 3.0.0, https://www.bioinformatics.nl/cgi-bin/primer3plus/primer3plus.cgi, accessed on 1 March 2023).

A single-step directional cloning strategy was employed to obtain knockout mutants. This involved cloning the upstream and downstream regions of the gene of interest (GOI) in the plasmid binary vector p507-HU2, which is based on the pRF-HU2 plasmid developed by Fransden et al. [37] but modified to be a high copy number plasmid (unpublished results). For overexpression mutants, a random integration into the fungal genome was carried out by a non-homologous recombination approach, cloning the gene of interest (GOI) into the plasmid p507-HUE, an in-house developed high copy number plasmid derived from the plasmid pRF-HUE developed by Frandsen et al. [37]. The amplification of the upstream and downstream regions and the GOIs from *A. carbonarius* AC49 genomic DNA (gDNA) was performed using a Phusion U Hot Start DNA polymerase (Thermo Scientific, Waltham, MA, USA, catalogue number F-555S) to ensure the fidelity of the reaction. The primer pairs dot1-O1/dot1-O2 and rmtA-O1/rmtA-O2 were used for amplifying the upstream regions of *dot1* (Gene ID:ASPCADRAFT_206324; Protein ID: OOF97488) and *rmtA* (Gene ID: ASPCADRAFT_205861; Protein ID: OOF97063) genes, respectively, while the primer pairs dot1-A3/dot1-A4 and rmtA-A3/rmtA-A4 were employed for amplifying the downstream regions of both genes, in the gene replacement strategy. Additionally, the primer pairs dot1-OE-A3/dot1-OE-A4 and rmtA-OE-A3/rmtA-OE-A4 were used for amplifying the GOI for the overexpression mutants (Table 2). Cycling conditions were 98 °C for 3 min, followed by 35 cycles of denaturation at 98 °C for 5 s, annealing at 61 °C for 10 s, extension at 72 °C for 1 min, and a last step of extension at 72 °C for 10 min.

The plasmids p507-H2-AcDot1, and p507-H2-AcRmtA were obtained through USER (Uracil-Specific Excision Reagent) friendly cloning technique by mixing upstream and downstream fragments with the digested vector p507-HU2 (at a ratio of 30:30:120 ng) and the USER mix (New England Biolabs, reference M5505S, Ipswich, MA, USA). Similarly, plasmids p507-HE-AcDot1 and p507-HE-AcRmtA were obtained using the same strategy, mixing the GOI fragment with the digested vector p507-HUE (at a ratio 30:120 ng) and USER mix. The USER reaction was initiated by keeping the mix at 37 °C for 20 min, followed by 20 min at 25 °C. After the reaction, an aliquot of 1 µL of the mixture was used for the transformation of chemically competent *E. coli* DH5α cells. This was achieved by keeping the mix on ice for 30 min followed by a thermal shock at 42 °C for 45 sec. After 1 h of incubation in LB medium at 200 rpm and 37 °C, an aliquot of 100 µL of cell suspension was used to inoculate LB agar medium supplemented with 25 µg∙mL^−1^ of kanamycin. After 18 h at 37 °C, resistant colonies were screened by PCR for the integration of the regions of interest. The primers pair RF-2/p507-HUE-Fw11 was used for the upstream region, and RF-1/RF-6, for the downstream region integration in the plasmids p507-H2. For the overexpression of the GOI in the plasmid p507-HUE, the primers pair RF-6/p507-HUE-Fw3 was used. The integrity of the GOI insertion and the absence of possible single-nucleotide polymorphism in the p507-HE-AcDot1 and p507-HE-AcRmtA plasmids were ensured by sequencing, performed by external service (Eurofins Genomics Europe Shared Services GmbH, Ebersberg, Germany).

The plasmids were integrated into *A. tumefaciens* AGL-1 electrocompetent cells using a Gene Pulser apparatus (Bio-Rad, Richmond, CA, USA). *A. carbonarius* mutants were obtained through *A. tumefaciens*-mediated transformation (ATMT) as described by Gerin et al. [68]. Monosporic cultures were derived from the hygromycin-resistant colonies, and the genomic DNA was extracted as described by Crespo-Sempere et al. [19].

The disruption of *dot1* and *rmtA* in the knockout mutants was confirmed by PCR amplification of the region between the upstream region and the hygromycin B resistance gene. The primer pairs dot1-1F/HPH-TER2 and rmtA-1F/HPH-TER2 were used for Δ*dot1* and Δ*rmtA* mutants, respectively. Amplification of the region between hygromycin B and the downstream region was conducted using the primers pairs dot1-2R/HPHPRO4 and rmtA-2R/HPHPRO4. Integration of the hygromycin resistance gene was verified using the primers Hyg-1R/Hyg-2F, and the disruption of the gene of interest with the primers dot1-3F/dot1-4R and rmtA-3F/rmtA-4R, for *dot1* and *rmtA*, respectively.

The number of T-DNA copies integrated into the genome was determined by qPCR as described by Gerin et al. [68], in a LightCycler 480 Instrument (Roche Diagnostics, Mannheim, Germany) equipped with the LightCycler SW 1.5 software (Roche Diagnostics) and by calculating the number of copies according to Pfaffl [69]. The primer pairs dot1-5F/dot1-6R and rmtA-5F/rmtA-6R, designed in the promoter region of the *dot1* and *rmtA* genes, were used for the knockout mutants while the couples of primers dot1-8F/dot1-4R and rmtA-8F/rmtA-4R for the OE mutants. The non-ribosomal peptide synthetase (*nrps*) gene (Gene ID: ASPCADRAFT_132610; Protein ID: OOF93601) was used with primers AcNRPS-F/AcNRPS-R as the reference gene.

### 5.4. Phenotypic Characterization and OTA Extraction

The growth, sporulation, and OTA production of three *A. carbonarius* mutant isolates per transformation were compared with the wild-type (WT) *A. carbonarius* AC49. For the evaluations of growth and OTA production, sterile 96-well plates containing 200 µL of PDB or MM were inoculated in triplicate with conidia suspension at a final concentration of 10^5^ conidia∙mL^−1^ (adapted from [70]). The plates were incubated under static conditions at 24 ± 1 °C, in the dark, with 80% humidity and the absorbance at 600 nm was automatically acquired every 2 h with a FLUOstar Omega (software version 6.20) microplate spectrophotometer (BMG Labtech, Ortenberg, Germany). After 6 days, the area under the curve (AUC) of each replicate was calculated in R4.1.1 [70,71], while OTA was extracted from each replicate with 250 µL of methanol in an Omni bead Ruptor 24 (Omni International Inc., Kennesaw, GE, USA) stirring the samples for 15 min with 30 s of shaking, at 3.10 speed, interspersed by 2 min. The samples were then centrifuged at max speed for 15 min and 300 µL of supernatant were recovered and filtered. At least 3 biological replicates were collected per isolate and stored at −20 °C until their use.

Evaluations on sporulation were conducted on 7-day-old PDA cultures, by resuspending conidia from 3 plugs (8 mm Ø) in 500 µL of 0.05% Tween 20 water and titrating the suspension in a hemocytometer.

To evaluate the germinability and germ tube elongation of the conidia, 10 µL of conidia suspension (1 × 10^5^ conidia∙mL^−1^) from one isolate per each transformation and WT were inoculated on agar glucose plugs (6 mm Ø) and incubated overnight at 24 ± 1 °C, under humidity saturation. For each isolate, the conidia germination and germ tube length of three technical replicates of 100 random conidia were observed under an optical microscope (Optiphot, Nikon Metrology NV, Tokyo, Japan). The observations on conidia morphology were conducted on conidia from 4-day-old PDA cultures, stained with calcofluor white, under an Eclipse 90i Nikon widefield microscope (Nikon Corporation, Tokyo, Japan) equipped with a 40× objective (CFI Plan Fluor DIC M/N240X) and a 5-megapixels cooled digital color camera (DS-5Mc, Nikon Corporation). Images were processed by the Nis Elements BR 3.2 software (Nikon Corporation) and with Fiji (ImageJ, version 1.49q, National Institutes of Health, Bethesda, MA, USA).

### 5.5. Gene Expression Study

A volume of 400 µL of conidia suspension (1 × 10^8^ conidia∙mL^−1^), obtained from one isolate per each transformation, were inoculated in triplicate in 100 mL Erlenmeyer flasks containing 40 mL of MM and incubated for 4 days in OTA-inducive conditions (OTA-I; 25 °C in the dark, without shaking). Total RNA was extracted from the mycelia, using the RNeasy Plant Mini Kit (Qiagen, Milan, Italy) and DNA digestion was performed with RQ1 RNase-Free DNase (Promega, Madison, WI, USA), according to the manufacturer’s indications. An aliquot of 20 µL of RNA was then purified with 100 µL phenol-chloroform (1:1) and precipitated with 10 µL of sodium acetate and 275 µL of ethanol absolute by freezing at −80 °C for 30 min. The pellet was then washed with 70% ethanol and resuspended in ultrapure water. The concentration and the quality of RNA were checked with Nanodrop™ 2000 spectrophotometer (Thermo Fisher Scientific Inc., Wilmington, DE, USA), and an electrophoresis separation with 1% agarose gel was performed to check the integrity. The cDNA was synthesized from 500 ng of RNA, in a volume of 20 µL using M-MLV Reverse Transcriptase (Thermo Fisher Scientific Inc.) following the manufacturer’s guidelines. The expression of the genes *otaR1* (JGI ID: 1075938), *otaA* (JGI ID:1051847), *otaB* (JGI ID:1155831), *otaC* (JGI ID:1128198), *otaD* (JGI ID:171871), *otaY* (JGI ID:1155830), *Acdot1* (JGI ID:681903) and *AcrmtA* (JGI ID:1105131) was determined in a real-time PCR Detection System CFX96TM (Bio-Rad Laboratories, Hercules CA, USA), using the primers reported in Table 2. Amplification reactions were conducted in 10 µL reaction mixture, containing 1x iQ^TM^ SYBR Green Supermix (Bio-Rad Laboratories), 100 nM of each primer and 1 µL of target cDNA, with the following cycling conditions: 3 min at 95 °C followed by 35 cycles of 10 s at 95 °C and 30 s at 60 °C. The no-retro transcript control qPCR was performed to ensure the lack of gDNA in the samples. Relative gene expression was calculated using the gene encoding ubiquitin (ID: 393986) as a reference gene, with CFX Manager^TM^ Software version 3.1 (Bio-Rad Laboratories) and the 2^−ΔΔCT^ method [72].

### 5.6. Response to Osmotic and Oxidative Stress

To investigate the effect of osmotic and oxidative stress on growth, sporulation, and OTA production of mutant isolates, a mycelial plug (6 mm Ø), obtained from 48 hours-old cultures was inoculated in triplicate in the center of Petri dishes (90 mm Ø) containing PDA supplied with 1 mM hydrogen peroxide or increasing concentrations of D-sorbitol (0.6, 1.2, 2.4, and 3.6 M) or NaCl (20, 40, 60, 80, and 100 g∙L^−1^). The plates were incubated for 7 days at 28 ± 1 °C, in the dark, and the growth was evaluated daily by measuring the orthogonal diameter of the colony. The same media without the addition of stress agents was used as control. Furthermore, at 7 days after inoculation (DAI) a conidia suspension in 0.05% Tween 20 was obtained from three plugs (8 mm Ø) and titrated in a hemocytometer to evaluate the sporulation. At the same time, OTA was extracted from 3 plugs (6 mm Ø), by adding 500 µL of methanol, vortexing for two minutes, and shaking at 200 rpm the samples for 2 h. OTA extracts were stored at −20 °C until HPLC analysis. The inhibition effect of each condition was calculated according to Abbot Index [73]: ((ULC-T)/ULC) × 100, where ULC is the value of mycelial growth, conidia production or OTA accumulation on the control medium and T is the value for the treatment.

### 5.7. In Vivo Assay

For each isolate, 5 µL of conidia suspension (1 × 10^4^ conidia∙mL^−1^), obtained as previous described from 7-day-old PDA cultures, were inoculated on the surface of three replicates of 4 grapes from 4 bunches of berries, previously sterilized in 2% sodium hypochlorite for 1 min, rinsed three times with sterile distilled water, airdried and wounded with a needle. Sterile distilled water instead of conidia suspension was used for the 12 berries used as control. The berries were incubated at 28 ± 1 °C in the dark humidity saturation and the area of the developing lesions was analyzed daily by Fiji (ImageJ 1.49q software, National Institutes of Health). The sporulation was evaluated 4 DAI on 3 replicates of 4 berries for each isolate, by washing the surface with 5 mL water supplemented by 0.05% of tween 20, collecting at least 98% of the spores. OTA was extracted from the same samples by OchraTest mycotoxin testing system (Vicam, Milford, MA, USA), following the manufacturer’s instructions.

### 5.8. OTA Quantification

OTA quantification was performed by HPLC analysis. A volume of 10 µL of filtered methanol extracts were injected into the chromatographic apparatus (ACQUITY Arc Sys Core 1–30 cm, Waters Co., Milford, CT, USA) equipped with a Kinetex^®^ reverse-phase C18 silica column (2.6 µm C18 100 Å, LC Column, dimensions 100 × 3 mm, Ea, Phenomenex Inc., Torrance, CA, USA) preceded by a KrudKatcher ULTRA HPLC In-Line Filter precolumn (2.0 µm Depth Filter × 0.004 in, Phenomenex Inc.) and a Waters 2475 fluorescence detector (330 nm excitation wavelength, 460 nm emission wavelength). A mobile phase of acetonitrile: water: acetic acid (57:41:2, *v*/*v*/*v*) with an isocratic elution of 6 min at flow rates of 0.30 mL × s^−1^ was used, and the extracts were identified by comparing the retention time with the OTA standard (Romer Labs Deutschland GmbH, Butzbach, Germany).

### 5.9. Statistics

ANOVA analysis, followed by Tukey’s Honest Significant Differences (HSD) or Tukey–Kramer test, was applied to confirm statistically significant differences with CoStat (version 4.451, CoHort Software, Monterey, CA, USA), at a significance level of *p* ≤ 0.05.

## Figures and Tables

**Table 1:** Number of T-DNA copies integrated into the genome of the mutants estimated by qPCR.

Strain	Genotype	Cq GOI	Cq NRPS	T-DNA Copies Estimated
AC49	Wild type	19.47 ± 0.15	19.62 ± 0.17	-
AcΔ*dot1-A5*	Knockout	21.61 ± 0.05	21.71 ± 0.05	1
AcΔ*dot1-A6*	Knockout	20.73 ± 0.11	21.09 ± 0.05	1
AcΔ*dot1-A10*	Knockout	21.33 ± 0.12	21.68 ± 0.04	1
AC49	Wild type	15.35 ± 0.12	15.94 ± 0.05	-
AcOE*dot1-B3*	Overexpression	19.82 ± 0.10	21.82 ± 0.06	3
AcOE*dot1-B6*	Overexpression	19.25 ± 0.12	21.82 ± 0.00	4
AcOE*dot1-B8*	Overexpression	19.31 ± 0.01	22.28 ± 0.07	5
AC49	Wild type	19.43 ± 0.06	19.47 ± 0.10	-
AcΔ*rmtA-C3*	Knockout	21.71 ± 0.03	21.40 ± 0.08	1
AcΔ*rmtA-C5*	Knockout	21.54 ± 0.04	21.14 ± 0.05	1
AcΔ*rmtA-C8*	Knockout	21.15 ± 0.11	21.14 ± 0.08	1
AC49	Wild type	16.23 ± 0.13	15.86 ± 0.05	-
AcOE*rmtA*-*D3*	Overexpression	21.44 ± 0.08	21.70 ± 0.07	2
AcOE*rmtA*-*D7*	Overexpression	20.58 ± 0.10	21.41 ± 0.13	2
AcOE*rmtA*-*D9*	Overexpression	19.91 ± 0.17	20.97 ± 0.04	3

**Table 2:** Principal features of the primers used in the work. Bold indicates the adaptors used for generation of 3′ single-stranded overhangs.

Name	Target	Sequence (5′-3′)	bp
Amplification of upstream region			
dot1-O1	*Acdot1* upstream	**GGTCTTAAU**CGAAACTACGGGCGAAGAAG	29
dot1-O2		**GGCATTAAU**GCGACGGTGAGAGGGAAA	27
rmtA-O1	*AcrmtA* upstream	**GGTCTTAAU**GTCTATTGGTTGGTTGGTTGCT	31
rmtA-O2		**GGCATTAAU**AGGTTAGCTGATTTGAACTTCCG	32
Amplification of downstream region			
dot1-A3	*Acdot1* downstream	**GGACTTAAU**CCCTAGCACTCTTCACATTCAG	31
dot1-A4		**GGGTTTAAU**TGGACGGTGAACTGAGCC	27
rmtA-A3	*AcrmtA* downstream	**GGACTTAAU**TCCGTTCGTGTATTAGCCGA	29
rmtA-A4		**GGGTTTAAU**ACAGGGCCAATCAAAGCTTG	29
CDS + 3′UTR amplification for overexpression			
dot1-OE-A3	*Acdot1*	**GGACTTAAU**ATGGGATTTTTCGACCACCT	29
dot1-OE-A4		**GGGTTTAAU**AGCACGCGCTGATATGTATG	29
rmtA-OE-A3	*AcrmtA*	**GGACTTAAU**ATGTCCGGGCAATCCGC	26
rmtA-OE-A4		**GGGTTTAAU**AGCGTCGACTCGGGGTATCTA	30
*E. coli* (DH5α) screening			
RF-2	p507-H2	TCTCCTTGCATGCACCATTCCTTG	24
p507-HUE-Fw11		CAAGAAAACGCCAGGAAAAG	20
RF-1	p507-H2	AAATTTTGTGCTCACCGCCTGGAC	24
RF-6		ACGCCAGGGTTTTCCCAGTC	20
RF-6	p507-HE	ACGCCAGGGTTTTCCCAGTC	20
p507-HUE-Fw3		TCAGTTCGAGCTTTCCCACT	20
Sequencing			
RF-3	p507-H2	TTGCGTCAGTCCAACATTTGTTGCCA	26
dot1-3F	*Acdot1*	GAGCACTTGCCCATTCCTCT	20
dot1-4R		CCACCAACATCGGTCCAACT	20
dot1-8F		GCTTTCACCCCTCAACTGAA	20
dot1-9R		GCCTCCTCATCGGGTATGTA	20
rmtA-3F	*AcrmtA*	AAGGCAGGTGCTAAGCATGT	20
rmtA-4R		ATCGCGCAGATAGAAGACGG	20
rmtA-8F		TGCATGCCATAAGCCTATCA	20
rmtA-9R		GACGACCTCTTCCATCTTGC	20
*A. carbonarius* mutants screening			
dot1-1F	Upstream *Acdot1*/*HygB*	GGTCAGGGACGTCATTGCA	19
HPH-TER2		GCTCCGTAACACCCAATAC	19
rmtA-1F	Upstream *AcrmtA*/*HygB*	GGCTGACTCCTCCGTACCTA	20
HPH-TER2		GCTCCGTAACACCCAATAC	19
dot1-2R	Downstream *Acdot1*/*HygB*	GGGAGGATGGGAAGTTGAGC	20
HPHPRO4		GCACCAAGCAGCAGATGATA	20
rmtA-2R	Downstream *AcrmtA*/*HygB*	GCCGCAATTCAGAGCTTGAC	20
HPHPRO4		GCACCAAGCAGCAGATGATA	20
hyg-1R	*HygB*	ATTTGTGTACGCCCGACAGT	20
hyg-2F		GATGTAGGAGGGCGTGGATA	20
dot1-3F	*Acdot1*	GAGCACTTGCCCATTCCTCT	20
dot1-4R		CCACCAACATCGGTCCAACT	20
rmtA-3F	*AcrmtA*	AAGGCAGGTGCTAAGCATGT	20
rmtA-4R		ATCGCGCAGATAGAAGACGG	20
T-DNA copy number			
dot1-5F	*Acdot1*	AAGGATTGACCCTCCAAGCG	20
dot1-6R		ATGAGCTGCCCTTGCCTTAG	20
rmtA-5F	*AcrmtA*	AGGCGGTTGACTTTCCAATG	20
rmtA-6R		TCAGCAAGGAGGAAGACGTAC	21
AcNRP-F	*nrps*	CTCCACCCATCCTCCCGTTC	20
AcNRP-R		AATCCATGTCCTCACCATCGC	21
Gene expression analysis			
AcbZIP-For	*otaR1* (*AcOTAbZIP*)	TTTCCCTAGGATCTCTCCTA	20
AcbZIP-Rev		TATTGGGGTCGGACAGGAAT	20
Acpks4-For	*otaA* (*AcOTApks*)	TCTGTATGAGCGCATCGCC	20
Acpks4-Rev		GCAGAAGGCCACTTTCCAG	20
AcotaYfor	*otaY* (*AcOTAcyc*)	ACCATCCTCACCACCCTTGT	
AcotaYrev		GGGACTCTGGGCTAACACCT	
Acnrps6-For	*otaB* (*AcOTAnrps*)	GATTCCGATGGAACTGCAAT	20
Acnrps6-Rev		CTGCCCCAGCATATCAATCT	20
AcP450-For	*otaC* (*AcOTAP450*)	GCCATACCTGACCGGGATCA	20
AcP450-Rev		GGGAAAATGGTCTCGTCGTG	20
Achal-For	*otaD* (*AcOTAhal*)	AAAGAAGCCTACACCGACTT	20
Achal-Rev		GAATTCGATGGATCCCGTGC	20
Acub-For	*Ubiquitin*	CCGAAGGTCAACTTCACCAC	20
Acub-Rev		GGCATATTTGCGAGTCCATT	20
*Acdot1*-For	*Acdot1*	TATTGGTCGAACAGCGTCAG	20
*Acdot1*-rev		AGGTTCATTGCATGCTTTCC	20
*AcrmtA*-For	*AcrmtA*	GGAAGTTCGCTTGTGTGGAT	20
*AcrmtA*-rev		GTACCGCGCCAACTGATAAT	20

## Data Availability

The data presented in this study are openly available in DIGITAL.CSIC at http://hdl.handle.net/10261/381243 (accessed on 10 February 2025).

## Supplementary Materials

The following supporting information can be downloaded at: https://www.mdpi.com/article/10.3390/toxins17040196/s1. Figure S1: Physical maps of the plasmids (A) p507-H2-AcDot1, (B) p507-H2-AcRmtA, (C) p507-HE-AcDot1, and (D) p507-HE-AcRmtA. (E) Diagram of the deletion cassette used to replace the target gene with the resistance marker (Hph) in the homologous recombination strategy for knockout mutants Δ*dot1* and Δ*rmtA* obtainment. (F) Diagram of the random integration strategy used to integrate the resistance marker and the gene of interest (GOI) preceded by the *PgpdA* in the genome of the overexpression mutants OE*dot1* and OE*rmtA*. Figure S2: Surface morphology of conidia from 4-day-old PDA cultures of (A) wild type, (B) Δ*dot1*, (C) OE*dot1*, (D) Δ*rmtA*, and (E) OE*rmtA*. The observation was conducted on conidia stained with calcofluor white through bright field (BF), RGB and epifluorescence (EF), with a 40× magnification. Figure S3: Germ tube elongation of conidia of WT and mutants isolates after overnight incubation at 24 °C on agar-glucose plugs (average length 300 µm).
