# Supplementary material for: Histone Methyltransferases AcDot1 and AcRmtA Are Involved in Growth Regulation, Secondary Metabolism, and Stress Response in Aspergillus carbonarius
Source: Toxins (Basel). 2025 Apr 12;17(4):196. doi: 10.3390/toxins17040196 (PMC12031602; doi:10.3390/toxins17040196)
Supplement: Supplementary file 1 [file toxins-17-00196-s001.zip › toxins-3512534-supplementary.pdf]

Supplementary Materials

Histone Methyltransferases AcDot1 and AcRmtA Are Involved in Growth Regulation, Secondary Metabolism, and Stress Response in *Aspergillus carbonarius*

Angelo Agnusdei [1], Adrián González-García [2], Donato Gerin [1,*], Stefania Pollastro [1], Francesco Faretra [1], Luis González-Candelas [2] and Ana-Rosa Ballester [2,*]

1 Department of Soil, Plant and Food Sciences, University of Bari Aldo Moro, Via Giovanni Amendola, 165/A, 70126 Bari, Italy; angelo.agnusdei@uniba.it (A.A.); stefania.pollastro@uniba.it (S.P.); francesco.faretra@uniba.it (F.F.)

2 Institute of Agrochemistry and Food Technology, Spanish Council for Scientific Research (IATA-CSIC), Calle Catedrático Agustín Escardino 7, 46980 Paterna, Valencia, Spain; adgongar@iata.csic.es (A.G.-G.); luis.gonzalez@iata.csic.es (L.G.-C.)

* Correspondence: donato.gerin@uniba.it (D.G.); ballesterar@iata.csic.es (A.-R.B.)

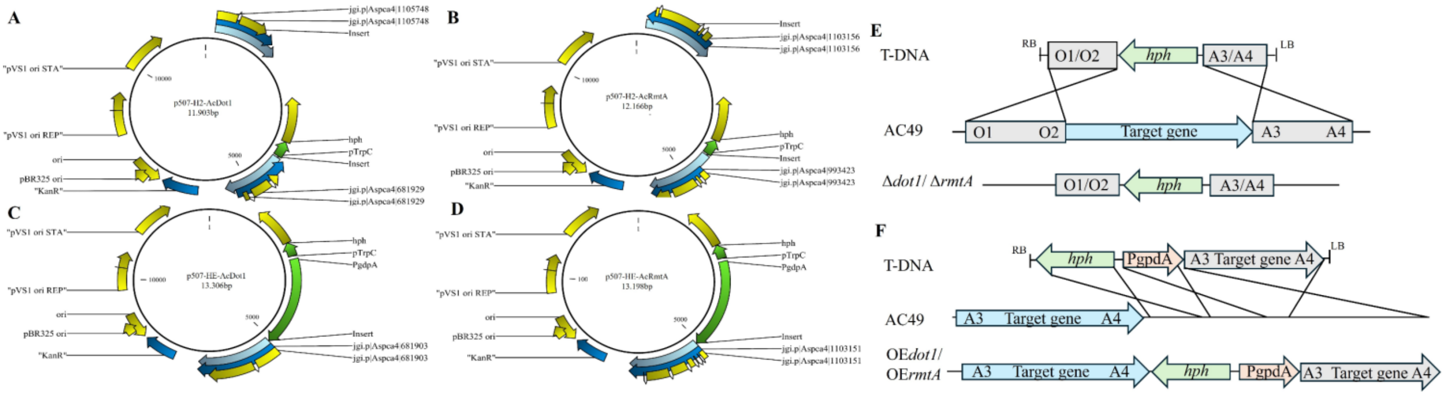

Figure S1: Physical maps of the plasmids A) p507-H2-AcDot1 B) p507-H2-AcRmtA C) p507-HE-AcDot1, and D) p507-HE-AcRmtA. E) Diagram of the deletion cassette used to replace the target gene with the resistance marker (Hph) in the homologous recombination strategy for knockout mutants Δ*dot1* and Δ*rmtA* obtainment. F) Diagram of the random integration strategy used to integrate the resistance marker and the gene of interest (GOI) preceded by the *PgpdA* in the genome of the overexpression mutants OE*dot1* and OE*rmtA*.

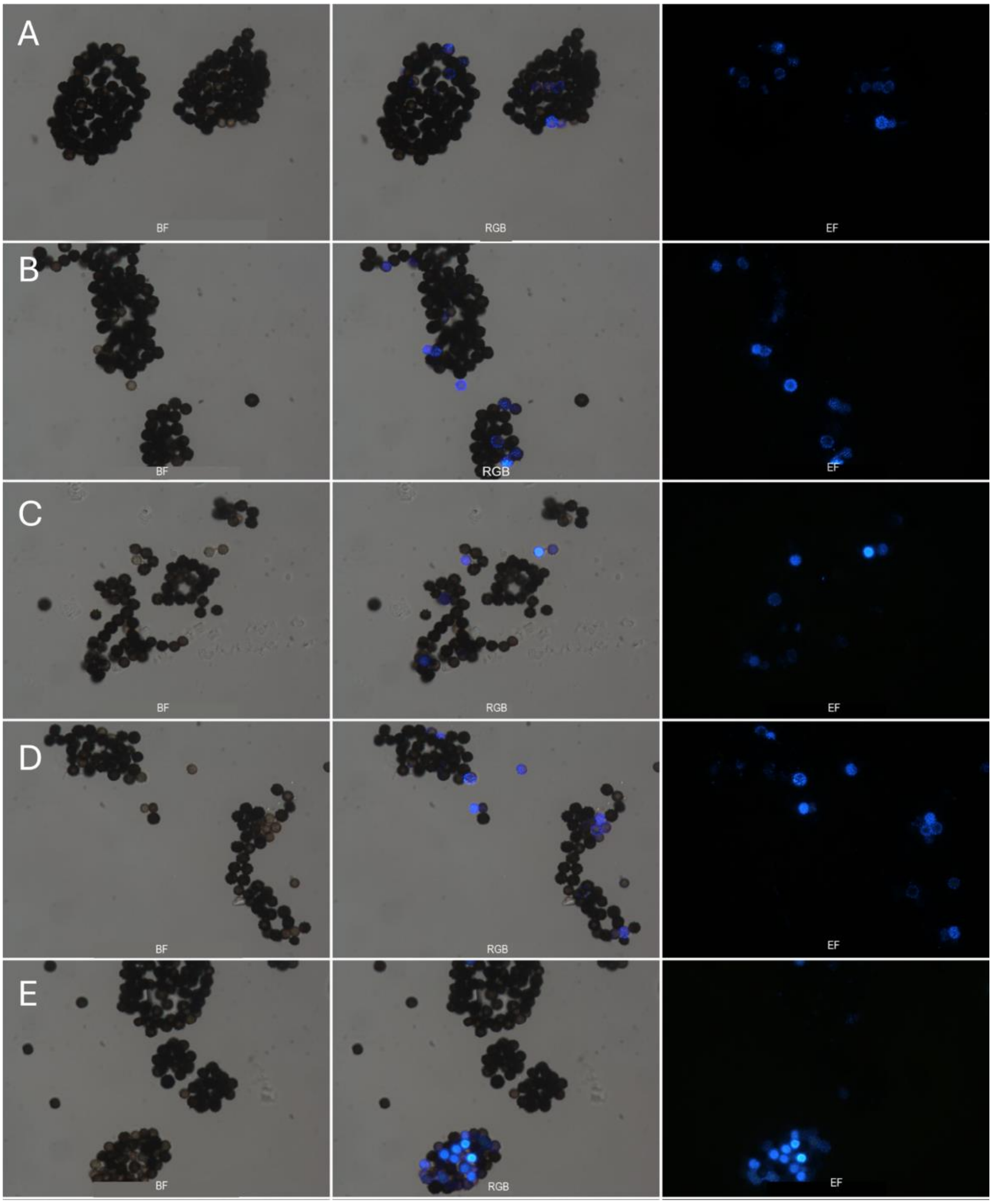

Figure S2: Surface morphology of conidia from 4 days-old PDA cultures of A) wild type B) Δ*dot1*; C) OE*dot1*, D) Δ*rmtA*, and E) OE*rmtA*. The observation was conducted on conidia stained with calcofluor white through bright field (BF), RGB and epifluorescence (EF), with a 40x magnification.

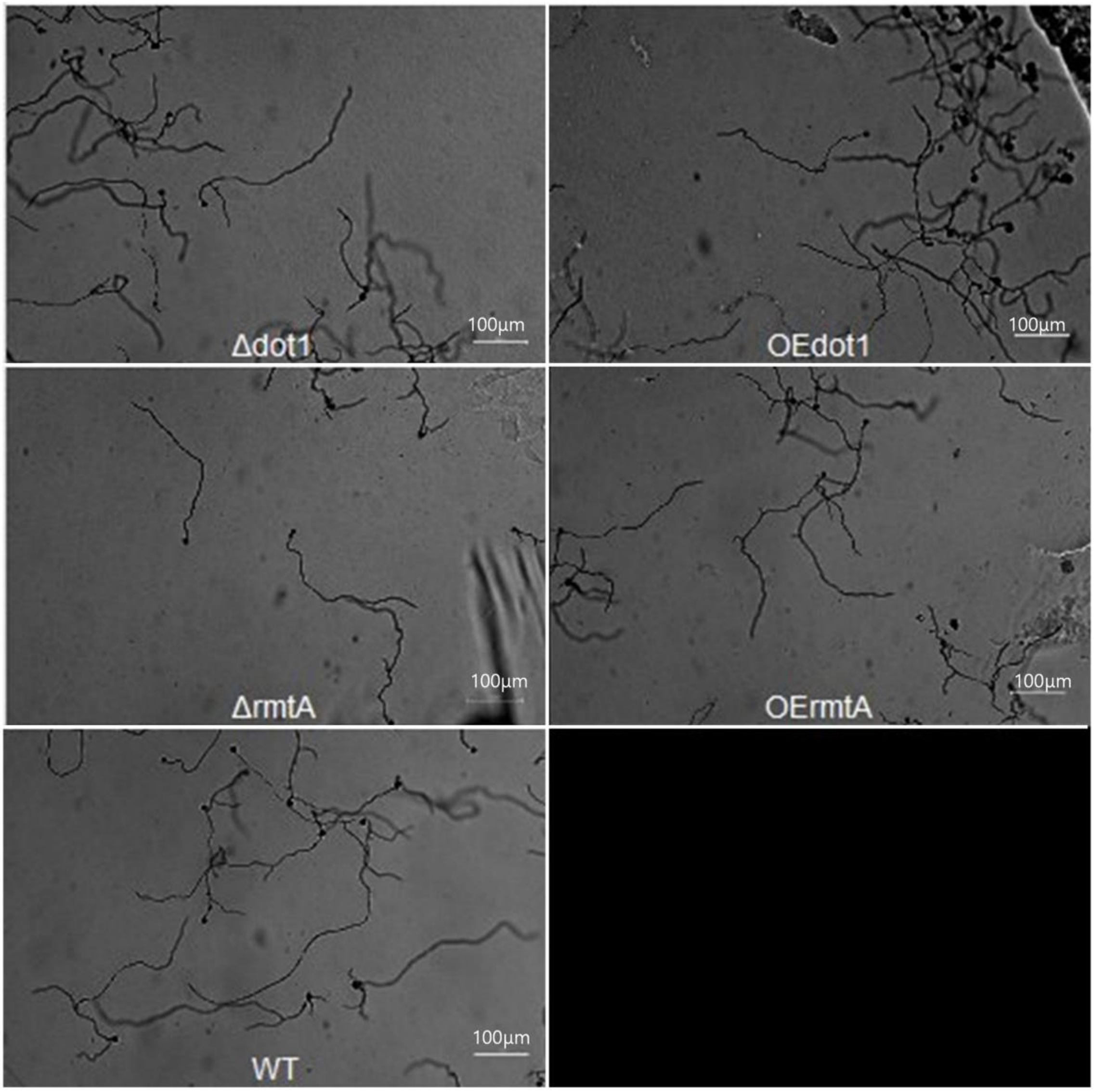

Figure S3: Germ tube elongation of conidia of WT and mutants isolates after overnight incubation at 24 °C on agar-glucose plugs (average length 300 µm).
